# Case report: One case of primary AL amyloidosis repeatedly misdiagnosed as scleroderma

**DOI:** 10.1097/MD.0000000000008771

**Published:** 2017-12-15

**Authors:** Linge Sun, Lei Zhang, Wenlu Hu, Tian-Fang Li, Shengyun Liu

**Affiliations:** Department of Rheumatology and Clinical Immunology, The First Affiliated Hospital of Zhengzhou University, Zhengzhou, China.

**Keywords:** amyloidosis, myeloma, scleroderma

## Abstract

**Introduction:**

Amyloid light chain (AL) results from the deposition of immunoglobulin light chain fragments, and can affect multiple organs/systems. Our patient was diagnosed as scleroderma repeatedly because of extensive skin thickening and hardening, but the treatment was not effective. We did extensive laboratory examinations including serum/urine protein electrophoresis and flow cytometry assay of bone marrow aspiration.

**Conclusion:**

A diagnosis of primary AL amyloidosis was established.

## 1 Introduction

The amyloidosis is a group of rare diseases caused by extracellular deposition of amyloid.^[1]^ It may affect multiple organs with protean manifestations, thus often causing delayed or incorrect diagnoses. While nephrotic syndrome, cardiomyopathy, and peripheral neuropathy are common in amyloidosis, it is relatively rare for a patient with the major presentations as myopathy and extensive skin involvement. Here, we report a case of a middle-aged woman who was hospitalized because of the difficulties in squatting and tongue movements, and scleroderma-like changes. This report had been approved by the Ethics committee of the First Affiliated Hospital of Zhengzhou University.

## 2 Case report

A 50-year-old female patient without the history of any diseases was admitted to our hospital in August 2016. She had difficulties in squatting for 5 years, impaired tongue movements, coarseness and dysphagia for 8 months, extensively tightened and pigmented skin for 6 months. She felt tingling when touching cold water and warming up hands relieved the symptom. The difficulty in squatting was noticed in year 2011 and the symptom kept progressing to the point where the skin became so extensively tightened that the movements of lower limbs were significantly restricted.

Constitutional symptoms included low fever, fatigue, joint pain, and morning stiffness. No Raynaud phenomenon and joint swelling were noticed. She once visited a physician at local hospital where she was diagnosed as scleroderma. She was treated with traditional Chinese medicines and very little improvement was achieved. Two years ago, she had snoring and apnea during sleep and was diagnosed as obstructive sleep apnea hypopnea syndrome, but she refused noninvasive ventilation. Eight months ago, she visited a neurologist in other hospital due to difficulty in opening mouth and moving tongue, dysphagia, hoarseness, and ever-worsening skin tightness. Brain magnetic resonance imaging (MRI) and magnetic resonance angiography (MRA) showed bilateral lacunar infarction of basal ganglia, white matter demyelination in mild cerebral, multiple sclerosis in cerebral arteries, and mild stenosis of bilateral posterior cerebral artery. Neuro-electrography revealed mild to moderate demyelinating peripheral neuropathy and the damage in the left pyramidal tract. Polysomnography revealed a moderate obstructive sleep apnea-hypopnea syndrome and mild hypoxemia. She was then diagnosed as extrapyramidal disease and sleep apnea-hypopnea syndrome, and treated with madopar tablet and neurotropin. However, no evident effect was observed.

Six months ago, she went to Beijing Union Hospital, the best hospital in China, because of flake skin pigmentation on her face, upper limbs, low back, and buttock areas. She was diagnosed as systemic sclerosis and treated with prednisone, Tripterygium glycosides, and methotrexate for 2 weeks. No improvement was achieved.

**Figure 1:**
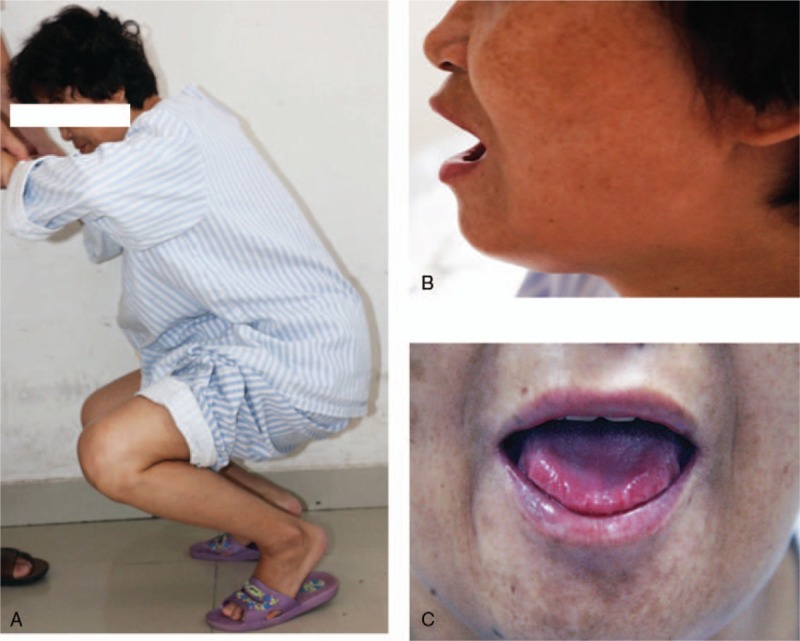
The patient had difficulty in full squatting (A) and mouth opening (B). The movements of tongue were limited, especially the extension. Macroglossia with slight lateral scalloping was noticed (C).

The patient was admitted to our hospital on August 24, 2016. Routine physicals showed normal vital signs. Extensive skin pigmentation was found in the above-mentioned areas. The skin became that so thickened and tightened that limb movements were seriously limited, especially when squatting (Fig. 1A). She had difficulty in opening the mouth (Fig. 1B). The tongue movements, especially, lolling, were significantly compromised. Macroglossia with lateral scalloping was noticed (Fig. 1C). She also complained of the difficulty in swallowing and hoarseness when talking. Hardening in bilateral mandible areas were noticed. Muscle strength was roughly at the Grade 4. No evident edema was found in the lower limbs. Extensive laboratory examinations were performed and the relevant results were listed as follows: IgG: 8.68 g/L (7.51–15.6), IgM: 0.9 g/L (0.46–3.04), IgA: 0.86 g/L (0.82–4.53), free light kappa chain: 6.24 g/L (6.29–13.5), lambda chain 3.42 g/L (3.13–7.23), and blood calcium concentration was 2.59 mM/L. Common autoantibodies related to the rheumatic diseases were all negative. The concentration of complement 3 (C3) was slightly low while that of C4 was normal.

A hardened nodule was found in submandibular area and ultrasound examination confirmed it was the thickened muscle in the base of the tongue. MRI examination of sublingual areas demonstrated abnormal signals in the bilateral epiglottis cartilage, laryngeal cavity, and the base of the tongue. The patient refused tongue biopsy. MRI of lower limbs showed a mild edema of subcutaneous soft tissue. Skeletal radiography did not detect apparent abnormality.

**Figure 2:**
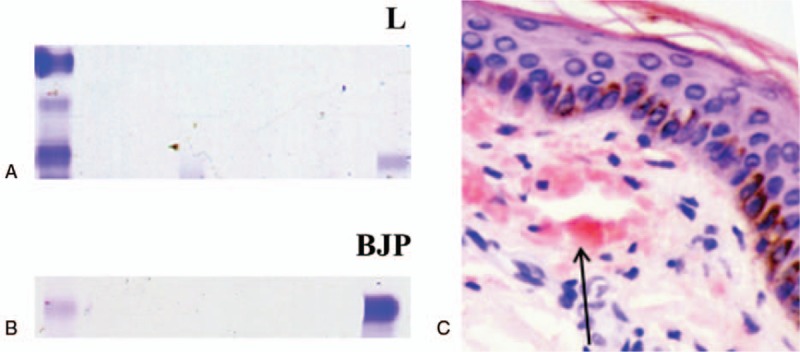
Serum protein eletrophoresis showed an evident band of Lamda chain (L) of immunoglobulin (A). The Bence–Jones Protein (BJP) was detected after urine electrophoresis (B). Histological examinations of skin biopsy specimens showed amorphous, Congo-red positive deposits (arrow) in the dermis (C).

Serum protein electrophoresis showed an apparent lambda light chain (Fig. 2A). Urine electrophoresis confirmed the existence of the Bence–Jones protein (Fig. 2B). Analysis of bone marrow specimens showed an increased percentage of plasma cells (12%) and immunostaining revealed a positive reactivity for a lambda light chain. Histological examination of skin biopsy samples showed amorphous, Congo red positive depositions in the dermis (Fig. 2C). A diagnosis of AL amyloidosis with skin and muscle involvement was then established. The patient was then referred to the Department of Hematology at our university hospital where she received chemotherapy consisting of velcade, cyclophosphamide, and dexamethasone. After 2 rounds of chemotherapy, skin thickening and hardening and dysphagia were significantly alleviated. Consistently, serum protein electrophoresis showed a decreased band of the lambda chain, and flow cytometry demonstrated a decreased percentage of plasma cells (8%).

## 3 Discussion

Monoclonal gammopathy (MG) of undetermined significance (MGUS) is an asymptomatic premalignant condition that can eventually progresses to a malignant plasma cell dyscrasia or lymphoproliferative disorder. MG with prominent skin involvements can be referred to MG of cutaneous significance (MGCS).^[2]^ Here, we report a case of primary AL amyloidosis mainly manifested as significant extensive skin involvement and muscle lesion. The presentations were so atypical that the patient was misdiagnosed repeatedly as scleroderma even by the most experienced physicians in China.

Macroglossia with slight lateral scalloping was an important sign that led us to investigate the possibility of AL amyloidosis. Other symptoms included difficulty in lolling and snoring, suggesting the pathologies in muscles. In addition, the difficulty in the movements of lower limbs, especially during squatting exercises, also indicates muscle involvement, although tightened skin may also play a role.

AL amyloidosis results from the deposition of fibrillar protein consisting of the light chain of immunoglobulin with the lambda type accounting for 75% of all cases.^[3]^ While this clonal B-cell disorder may be associated with multiple myeloma (MM) and lymphoma, it can also be idiopathic.^[4]^ Clinical and laboratory data, for example, plasmacytosis (12%) from bone marrow assay, indicate that the original disease is (MM). Amyloidosis occurs in about 15% of MM patients.^[5]^ Protean manifestations often lead to delayed and incorrect diagnosis. Biopsy specimens with positive Congo red staining is critical for its diagnosis. Identification of the type of immunoglobulin light chains helps unveil the underlying diseases.^[6]^ Although cumbersome, mass spectrometry of amyloid material remains the gold standard for its final diagnosis.^[7–10]^

Cutaneous and mucous involvements have been reported by different research groups and the major manifestations are summarized in Table 1.^[11]^ Although scleroderma-like changes have also been reported before (Table 2),^[12–20]^ extensive cutaneous involvement seen in our patient is not common. Severe hardening and pigmentation led to an incorrect diagnosis as scleroderma repeatedly. After thorough examinations and discussions, we excluded the possibility of scleroderma because of the presence of the presence of microglossia and the absence of Raynaud phenomenon, and the anti-Scl-70 and ACA antibodies. In addition, low dose of glucocorticoid together with weekly use of methotrexate did not show any effect. The diagnosis of POEMS could not be established because of atypical clinical manifestations and the absence of evident angiogenesis up histological examination. After serum and urine electrophoresis and analysis of the skin and bone marrow specimens, a primary AL amyloidosis was established, which was consolidated by prompt response to chemotherapy.

**Table 1:**
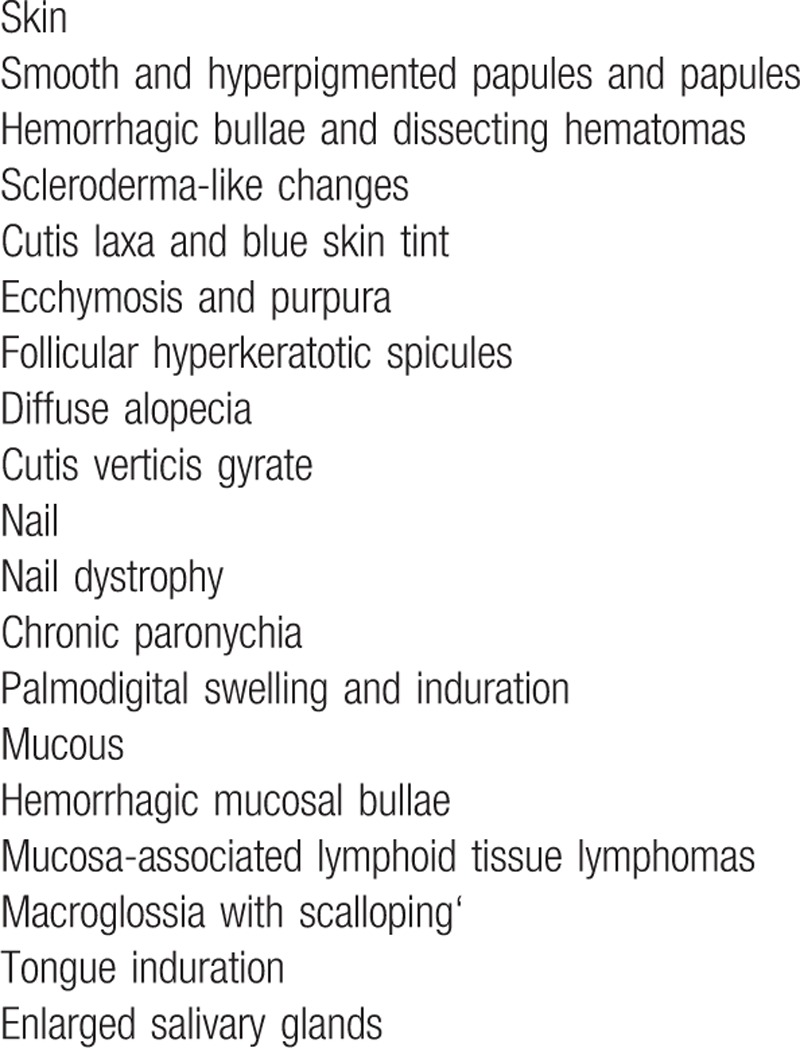
Skin, nail, and mucous manifestations.

**Table 2:**
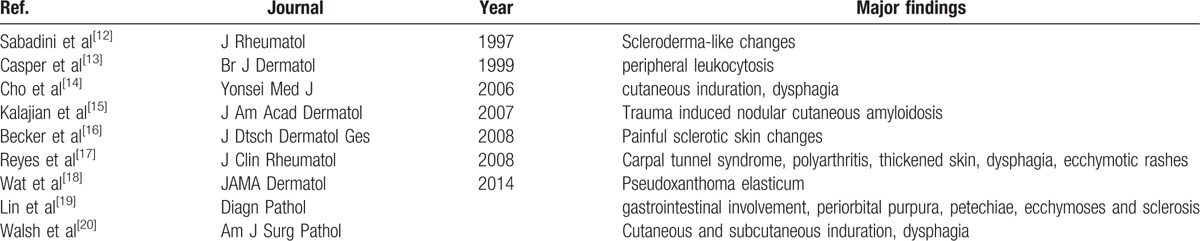
Scleroderma-like changes in primary amyloidosis.

The treatment of AL amyloidosis is similar to that of MM,^[21]^ with the ultimate goal being the eradication of monoclonal plasma cells in bone marrow and prevention of production of pathological immunoglobulin light chains. We hope that this report will help our peers keep vigilant of underlying diseases when similar clinical scenario occurs in order to make a prompt diagnosis and effective treatment.
